# Rapid Discrimination of Neuromyelitis Optica Spectrum Disorder and Multiple Sclerosis Using Machine Learning on Infrared Spectra of Sera

**DOI:** 10.3390/ijms23052791

**Published:** 2022-03-03

**Authors:** Youssef El Khoury, Marie Gebelin, Jérôme de Sèze, Christine Patte-Mensah, Gilles Marcou, Alexandre Varnek, Ayikoé-Guy Mensah-Nyagan, Petra Hellwig, Nicolas Collongues

**Affiliations:** 1 Laboratory of Bioelectrochemistry and Spectroscopy, UMR 7140, University of Strasbourg, CNRS, 4 Rue Blaise Pascal, 67000 Strasbourg, France; marie.gebelin@etu.unistra.fr (M.G.); hellwig@unistra.fr (P.H.); 2 Biopathology of Myelin, Neuroprotection and Therapeutic Strategies, INSERM U1119, Federation of Translational Medicine of Strasbourg, Université of Strasbourg. 1, Rue Eugène Boeckel, 67000 Strasbourg, France; Jerome.deseze@chru-strasbourg.fr (J.d.S.); cmensah@unistra.fr (C.P.-M.); gmensah@unistra.fr (A.-G.M.-N.); 3 Department of Neurology, University Hospital of Strasbourg, 1 Avenue Molière, 67200 Strasbourg, France; 4 Laboratory of Chemoinformatics, UMR 7140, University of Strasbourg, CNRS, 4 Rue Blaise Pascal, 67000 Strasbourg, France; g.marcou@unistra.fr (G.M.); varnek@unistra.fr (A.V.); 5 Department of Pharmacology, Addictology, Toxicology and Therapeutic, University of Strasbourg, 67000 Strasbourg, France

**Keywords:** machine learning, diagnosis, infrared spectroscopy, neuromyelitis optica spectrum disorder, multiple sclerosis

## Abstract

Neuromyelitis optica spectrum disorder (NMOSD) and multiple sclerosis (MS) are both autoimmune inflammatory and demyelinating diseases of the central nervous system. NMOSD is a highly disabling disease and rapid introduction of the appropriate treatment at the acute phase is crucial to prevent sequelae. Specific criteria were established in 2015 and provide keys to distinguish NMOSD and MS. One of the most reliable criteria for NMOSD diagnosis is detection in patient’s serum of an antibody that attacks the water channel aquaporin-4 (AQP-4). Another target in NMOSD is myelin oligodendrocyte glycoprotein (MOG), delineating a new spectrum of diseases called MOG-associated diseases. Lastly, patients with NMOSD can be negative for both AQP-4 and MOG antibodies. At disease onset, NMOSD symptoms are very similar to MS symptoms from a clinical and radiological perspective. Thus, at first episode, given the urgency of starting the anti-inflammatory treatment, there is an unmet need to differentiate NMOSD subtypes from MS. Here, we used Fourier transform infrared spectroscopy in combination with a machine learning algorithm with the aim of distinguishing the infrared signatures of sera of a first episode of NMOSD from those of a first episode of relapsing-remitting MS, as well as from those of healthy subjects and patients with chronic inflammatory demyelinating polyneuropathy. Our results showed that NMOSD patients were distinguished from MS patients and healthy subjects with a sensitivity of 100% and a specificity of 100%. We also discuss the distinction between the different NMOSD serostatuses. The coupling of infrared spectroscopy of sera to machine learning is a promising cost-effective, rapid and reliable differential diagnosis tool capable of helping to gain valuable time in patients’ treatment.

## 1. Introduction

Neuromyelitis optica spectrum disorder (NMOSD) and relapsing-remitting multiple sclerosis (RRMS) are autoimmune inflammatory and demyelinating diseases of the central nervous system. Because they can both share various clinical and radiological features, NMOSD has long been considered a variant of MS. However, the discovery of a specific antibody against the water channel aquaporin-4 (AQP4-Ab) in the serum of NMOSD patients led to successive diagnostic criteria being proposed since 2006 [1,2]. The diagnostic criteria revised in 2015 [2] were built around AQP4-Ab as a key marker that became sufficient for NMOSD diagnosis.

To date, the most accurate assay for AQP4-Ab detection is a cell-based assay using living cells that provides a sensitivity of 74.4% and a specificity of near 100% [3]. Around 20% of patients with NMOSD are seronegative for AQP4-Ab and some have an antibody against the myelin oligodendrocyte glycoprotein (MOG-Ab), a glycoprotein present at the surface of the myelin sheath [4]. For the detection of MOG-Ab in serum, strict recommendations have been provided to limit the rate of false results, but, despite high standards of quality, the sensitivity and specificity remain relatively low [5,6]. Finally, MOG-IgG serum concentration depends on disease activity and treatment status [7]. Around 18% of all NMOSD patients have neither antibody, i.e., neither AQP4-Ab nor MOG-Ab [8], and are generally referred to as double seronegative (DN) NMOSD patients. Of particular note is the fact that these DN patients share some clinical features and therapeutic response with AQP4-Ab-positive patients [9].

A critical point for early diagnosis is that, contrary to RRMS, disability in NMOSD is a direct consequence of the relapses [10,11]. The management of NMOSD is based on immunosuppressive drugs that are mostly not used in RRMS, whereas treatments used in RRMS can increase disability in NMOSD [12]. Thus, despite wide access to serological antibody testing for clinicians in practice, the delay to the results and the variety/variability of the tests could impair the management of treatment at the acute phase of the disease. Furthermore, accurate and rapid distinction between NMOSD and RRMS is still challenging due to the diversity of phenotypes of both diseases and the similarity of their symptoms and clinical features [13]. For instance, DN NMOSD patients are at risk of being overlooked or of being misdiagnosed with RRMS.

The disease course of MOG-ab-positive NMOSD patients differs from that of other NMOSD patients [14]. Therefore, MOG-ab-positive NMOSD patients require suitably adapted disease management, including rapid introduction of treatment. In addition, the accurate detection of MOG-Ab is still challenging [6], and MOG-Ab can be extremely rarely detected in MS [15]. Thus, there is an unmet need for a rapid and unambiguous diagnosis of MOG-ab-positive NMOSD patients as early as possible. In order to improve the accuracy and speed of diagnosis, additional highly-sensitive and highly-specific approaches are needed.

Fourier-transform infrared (FTIR) spectroscopy is a sensitive, rapid and cost-effective analytical tool. We have previously shown that the technique can be used as a highly efficient tool to discriminate between sera samples of healthy control (HC) subjects, progressive MS patients and amyotrophic lateral sclerosis patients [16]. Here, we used FTIR spectroscopy coupled to a random forest classifier [17], a machine learning algorithm, as a tool to discriminate between the different sera samples. To this end, we built a model based on sera samples from NMOSD and RRMS patients vs. sera samples from non-NMOSD subjects and non-RRMS patients. NMOSD and RRMS samples were collected after a first relapse in naïve patients. The negative instances comprised sera from healthy subjects (HC) and sera from patients suffering from peripheral neuropathies (NEUR), i.e., chronic inflammatory demyelinating polyneuropathy, an autoimmune disease resulting from damage to the myelin sheath of the peripheral nerves. The main result of this study is a diagnostic procedure discriminating between HC, NMOSD patients, RRMS patients and NEUR patients, regardless of the serostatus of the NMOSD patients. This new tool can reinforce the existing diagnostic protocols, thereby reducing the risk of misdiagnosis. Moreover, the development of this approach as a point-of-care diagnostic tool can considerably shorten the diagnosis time because the results can be obtained within minutes. We also report our efforts to discriminate NMOSD patients according to their serostatus. Finally, we analyzed in detail the question of distinguishing RRMS patients from NMOSD patients according to the serostatus of the latter.

## 2. Results

### 2.1. HC vs. NMOSD vs. RRMS vs. NEUR

**Figure 1:**
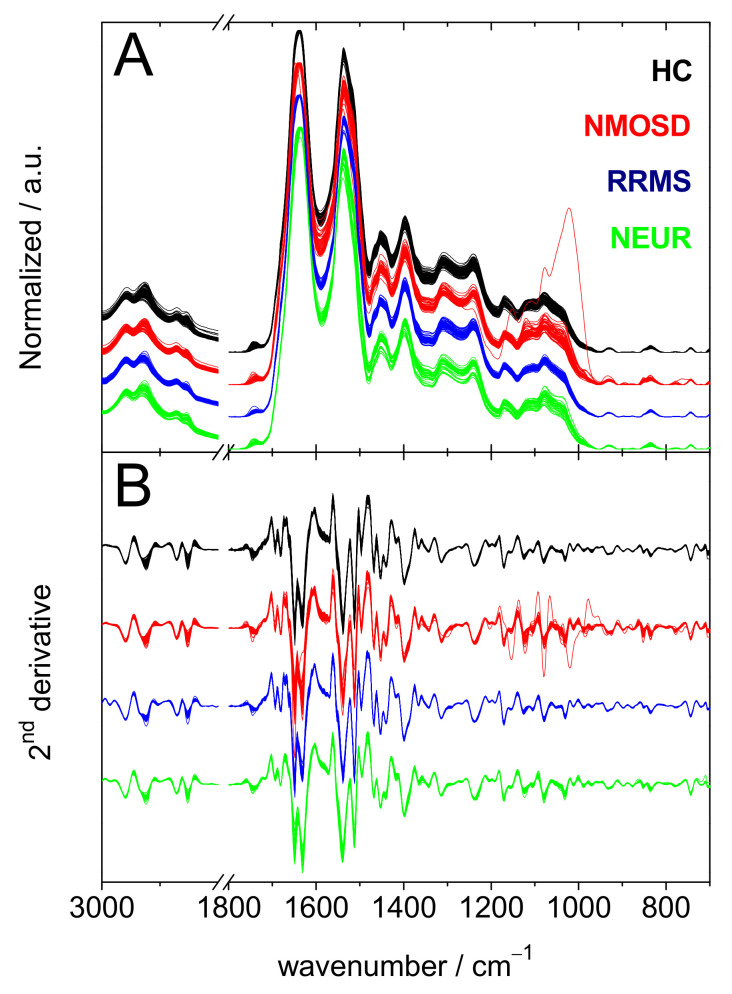
Infrared data of the 235 serum samples included in this study. (**A**) Normalized Fourier-transform infrared (FTIR) spectra of all serum samples in the 3000–2800 and 1800–700 cm^−1^ spectral ranges. (**B**) Second derivatives of the spectra of panel A. Healthy controls (black), neuromyelitis optica spectrum disorder (NMOSD) (red), relapsing-remitting multiple sclerosis (RRMS) (blue) and peripheral neuropathies (NEUR) (green). The groups of spectra and derivatives are off-set for clarity.

FTIR spectra of serum samples from all subjects (Figure 1) show bands dominated by the contributions from proteins, lipids and other biomolecules [18]. One spectrum of serum from an NMOSD patient had distinct intense signals in the 1185–950 cm^−1^ spectral range; this particular spectrum will be discussed later (see below). Most likely, these signals arise from glucose [19], despite the fact that other biomolecules are known to contribute in this spectral range [20,21,22]. Table S1 of the Supporting Information provides tentative assignments of the peaks found in the average spectra of each subject group (Figure S1).

Based on the second derivatives of the spectra, a classification model was built using 208 out of the 235 derivatives as a training set. The remaining 27 derivatives constituted an external validation set. Table 1 summarizes the performances of the random forest machine learning algorithm in discriminating the pathologies according to the spectra of the sera. The detailed count of successes and errors is reported in the “Classified as” column. The remaining columns contain the performances of the random forest model when it was used to discriminate one class against all the others. In a nutshell, the sensitivity and specificity measure the ability of the model to identify the true positives and the true negatives, respectively; the precision is the proportion of true positives among those instances identified as positive by the model; the area under the receiver operating characteristic curve (ROC AUC) measures the capacity of the model to rank higher positive instances compared to negative ones—a 100% value means that the ranking is perfect and 50% means that it is random.

The upper part of Table 1 reports the two-fold cross-validation results obtained on the training set: (i) a randomized half of the training data was used for fitting the parameters of the model; (ii) the model was then used to predict the pathologies on the other half; (iii) the role of those subsets was finally exchanged. To avoid being biased by a particular division of the training set into two halves, the procedure was iterated 10 times and the mean and standard deviation are also shown in Table 1. The lower part of the table reports the performances of the model trained on the entire training set and applied to the validation set. The validation set was kept isolated from the training set and was used for this sole purpose.

**Figure 2:**
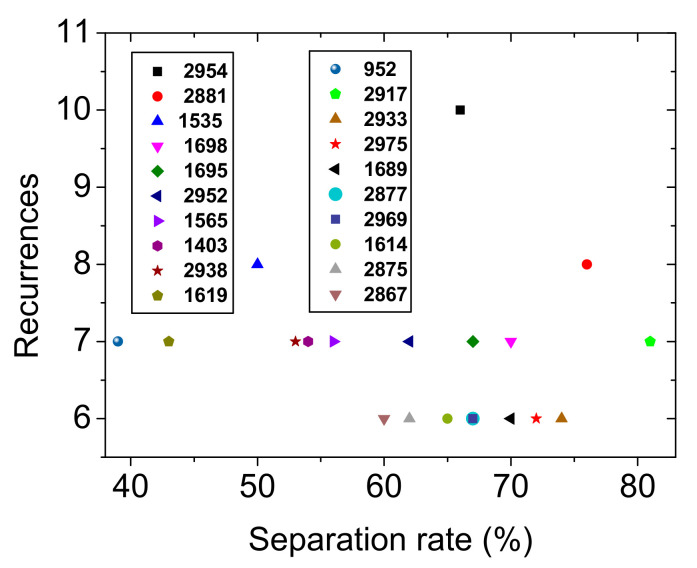
Scatterplot of the top 20 nodes with the highest recurrence in the random forest model of Table 1 and their corresponding percentage of separation rate.

The top 20 wavenumbers used by the model to distinguish the various serostatuses are shown in Figure 2. The separation rate is actually an average impurity decrease; when a given frequency is used in one of the random trees of the random forest, it describes how efficient it is to split the instances according to the group of sera. The recurrences are the number of nodes in all random trees of the random forest that are using the frequency.

To estimate how variations in the population of classes may impact the performances, we added in the Supporting Information (Table S2) additional results where the proportion of instances in each class in the test set is controlled, using a resampling technique. We did not observe significant changes in the performances of the models whether the model was trained using a unform distribution of samples in the classes (i.e., 30 samples in each class) or on the original data set.

### 2.2. AQP-4 vs. MOG. vs. DN

**Figure 3:**
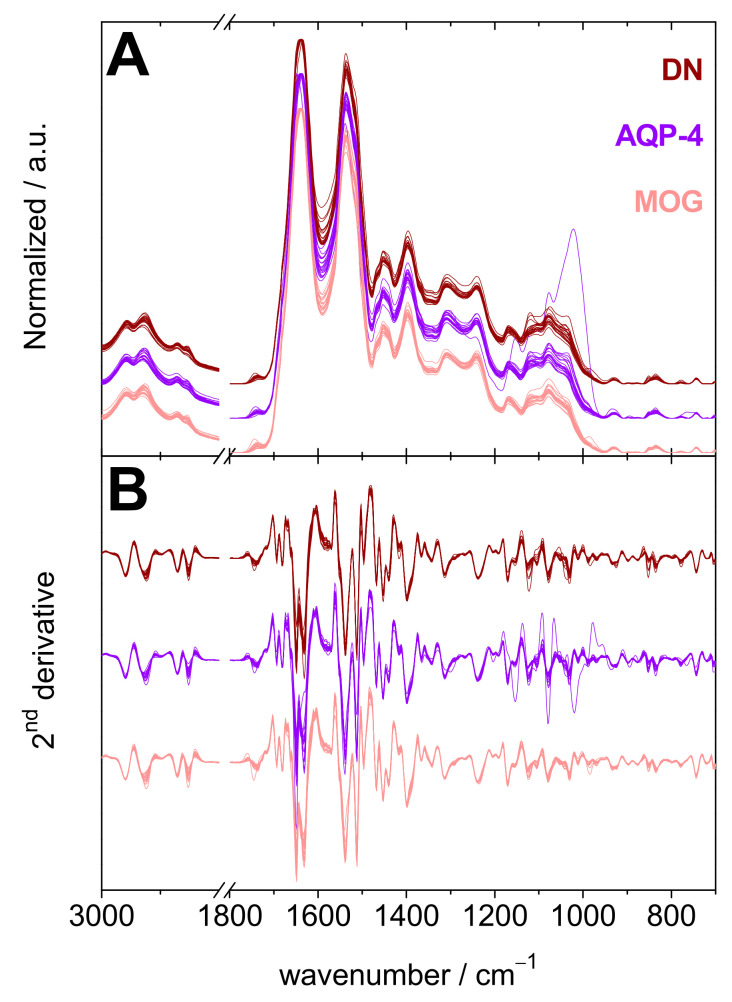
Infrared data of NMOSD sera samples. (**A**) Normalized FTIR spectra of the sera samples of all NMOSD patients in the 3000–2800 and 1800–700 cm^−1^ spectral ranges. (**B**) Second derivatives of the spectra of panel A. Double negative (DN) (dark red), AQP-4-Ab-positive (AQP-4) (violet), and Myelin oligodendrocyte glycoprotein-Ab-positive (MOG) (light red). The groups of spectra and derivatives are off-set for clarity.

**Figure 4:**
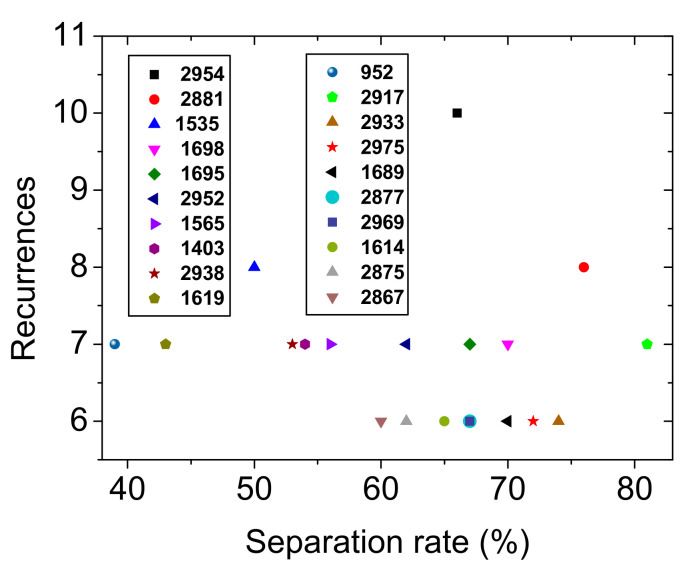
Scatterplot of the top 20 nodes with the highest recurrence in the random forest model of Table 2 and their corresponding percentage of separation rate.

The 60 sera samples of NMOSD patients participating in this study had one of the three serostatuses; AQP-4-Ab-positive (referred to here as AQP-4), MOG-Ab-positive (referred to here as MOG) or double negative for both AQP-4-Ab and MOG-Ab (referred to here as DN) are compared to each other in Figure 3, which shows the different classes of spectra (Panel A), and their second derivatives (Panel B). By using the second derivatives of 54 spectra of the three phenotype NMOSD spectra, a classification model was built and was tested on the six second derivatives not utilized to build the model. The resulting statistical analysis is summarized in Table 2 and the top 20 wavenumbers used by the model to distinguish the various serostatuses are shown in Figure 4.

### 2.3. RRMS vs. DN, RRMS vs. MOG and RRMS vs. AQP-4

NMOSD and RRMS patients can be difficult to differentiate, in particular when disease markers (radiological and serological) are elusive. In order to tackle the problem of misdiagnosis and to offer a faster differentiation between NMOSD and RRMS patients, we built three models based on the second derivatives of RRMS shown in Figure 1 and those of NMOSD shown in Figure 3. Each model contains 72 derivatives (54 of RRMS and 18 of each of the three NMOSD serostatuses). These models were tested on validation sets containing six derivatives of RRMS and two derivatives of each of the three NMOSD serostatuses. The resulting confusion matrices and performance figures are presented in Table 3.

## 3. Discussion

Here, we demonstrate that the challenging differential diagnosis or RRMS and NMOSD in the clinic can be made easily and quickly with the help of the random forest model built on the infrared signature of patients’ sera samples. In addition, NMOSD patients positive for anti-MOG antibody can rapidly and easily be distinguished from RRMS patients. Thus, disease management can be improved through the timely use of the appropriate treatment, thereby improving patients’ quality of life.

The use of infrared spectroscopy to distinguish diseases has been growing over the last few decades. Several proof-of-principal studies have been published [16,23,24,25,26,27], and literature reviews are also available [28,29,30,31]. The performance of the random forest models was estimated using two-fold cross-validation to discriminate between NMOSD, RRMS, NEUR and HC (see Table 1).

Two out of the seventy HC samples were misclassified as NMOSD; one out of the fifty-four RRMS samples and one out of the fifty-four NMOSD samples were misclassified as HC. Four clinically relevant statistical measures were computed for each class. First, the ROC AUC measures the probability that a random positive instance is ranked before a random negative one [32]. The ROC curves for the four classes shown in Figure S2 were higher than 97%, which reflects an extremely low number of missed true positive values for no cost in terms of false positives. To check the robustness of the reported statistics, we repeated the cross-validation procedure 10 times with random attribution of the instances to the cross-validation folds. These 10 iterations yielded very high ROC AUC values and very low standard deviation from the mean value (See Table 1). For instance, for NMOSD and RRMS, the ROC AUC values were 98.7 ± 0.9 and 100 ± 0.0%, respectively. The very high ROC areas and the low standard deviation from the mean value show that it is possible to adjust the threshold of the model to retrieve more true positive occurrences of a particular class at the cost of misclassifying a few more true positive occurrences of another class. For example, the specificity towards NMOSD (78.3 ± 7.3%) is lower than that towards the three other classes and the false negative rate is higher. Because of the high ROC AUC values, it is possible to adjust the model to lower the risk of overlooking NMOSD patients.

Testing the model built with the training set on an unknown validation set yielded a perfect discrimination rate between the four pathology groups (i.e., HC, NMOSD, RRMS and NEUR), which means that the risk of misdiagnosis was too low to be measured. The importance of this result lies in the fact that RRMS and NMOSD are challenging to diagnose in the clinic due to the similarity in symptoms. In addition, DN patients have a higher risk of being misdiagnosed with RRMS due to the absence of known biomarkers in the serum [33].

In addition, all HC were correctly classified and the risk of diagnosing a healthy subject with either disease was low. Lastly, the model did not confuse NEUR patients with either NMOSD or RRMS patients or with HC subjects, proving the very high discrimination rate between the autoimmune inflammatory diseases (NMOSD and RRMS) and the similar, yet different, autoimmune NEUR affecting the peripheral nervous system. The model relies on cornerstone spectral markers to distinguish various pathologies. These markers are identified in Figure 2 where the separation rates of the top 20 most frequently recurring wavenumbers in various decision trees are shown. Out of these 20 different wavenumbers, 15 were between 2977 and 2881 cm^−1^, which is a spectral range dominated by the absorption of the antisymmetric stretching vibration of CH_3_ and CH_2_ groups. Thus, the data suggest that a major difference in the composition of lipids and/or lipoproteins is to a large extent responsible for the distinction of various samples. Moreover, four different wavenumbers were in the 1695–1523 cm^−1^ spectral range, which is mostly dominated by the amide I and II modes of proteins. Finally, 1403 cm^−1^ also appears among the top 20 wavenumbers used in the model. This signal may have arisen from the deformation vibration of CH_2_ of lipids.

One of the NMOSD samples showed much higher intensities for the absorption bands in the 1185–950 cm^−1^ spectral range compared to the other serum samples (Figure 1). This spectral range is dominated by contributions from carbohydrates [19] and most likely DNA and RNA [20,21,22]. The contribution of these biomolecules to the distinction of NMOSD from the other pathologies was not significant since the top 20 nodes occurring in the random forest model do not include any signal below 1200 cm^−1^.

The focus then shifted towards the distinction of NMOSD patients based on their respective serostatus. For this purpose, another random forest model was built using the second derivatives of the infrared spectra (Figure 3) of sera of the three serostatuses of NMOSD patients. The performances measured using two-fold cross-validation and on an independent validation set are summarized in Table 2. The ROC curves are shown in Figure S3. The model showed a modest performance in discriminating the NMOSD patients based on their serostatus. The highest ROC AUC value obtained from 10 iterations of the model was for DN and was equal to 69.4 ± 6.6%. This is indicative that there was no clear signal discriminating the patients according to their serostatus. Most likely, there was no obvious biomarker in the sera for this task and the number of readily available instances was too low to identify some specific IR signal. For instance, both anti-AQP-4 and anti-MOG antibodies are proteins and the difference in their spectroscopic signature is likely too small to be picked up by the random forest model. Moreover, sample collection from DN patients took place a relatively long time after the relapse (See Table S3), compared to AQP-4 and MOG patients; thus, the absence of AQP4-Ab and MOG-Ab in DN patients just after the relapse most likely led clinicians away from an NMOSD diagnosis. Later on, when the NMOSD diagnosis was confirmed, the concentration of antibodies was too low to be detected. Accordingly, DN patients could in reality be either AQP4 or MOG-positive, which would explain the higher confusion rate observed among the NMOSD subtypes. The pathophysiology of DN NMOSD remains elusive but some explanations could lie in the variation of Ab levels throughout the disease that leads to a disappearance of Ab over time. Despite this possible phenomenon, a dominant humoral process remains the basis of the immunological mechanism that could be involved and makes it possible to clearly distinguish this population from patients with RRMS.

The performance of each of the three random forest models shown in Table 3 clearly demonstrates that sera of RRMS patients can easily be distinguished from those of NMOSD patients positive for AQP4-Ab or MOG-Ab or negative for both antibodies. With 10 iterations, the ROC AUC (Figure S4) values were very high in the three models, with the lowest value found for AQP-4 equal to 99.6%. Thus, the models are extremely performant and allow excellent differentiation between RRMS and any NMOSD serostatus. Accordingly, the risk of misdiagnosis of either serostatus of NMOSD with RRMS patients is extremely low. This spectroscopic approach combined with a machine learning algorithm is a rapid and reliable way of making a differential diagnosis that can be very useful in the clinic. In fact, the recording of an FTIR spectrum and the application of the machine learning model takes less than half an hour, whereas the analysis of serum biopsies can take weeks. Thus, the approach presented here can help to gain valuable time in the treatment of patients.

## 4. Materials and Methods

**Figure 5:**
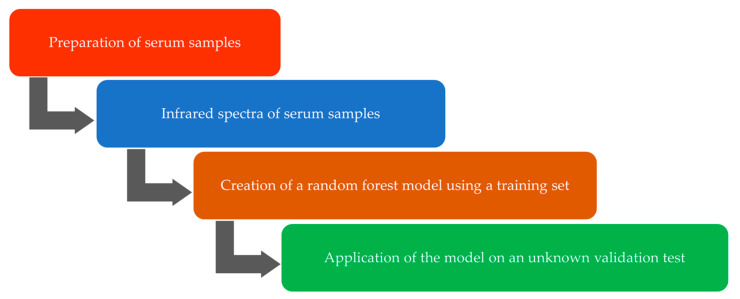
Workflow diagram showing the successive steps towards the distinction of the sera samples of the patients.

An overview of the workflow conducted to achieve the distinction between the serum groups is depicted in Figure 5. Details about all the workflow sections are presented below.

### 4.1. Sample Preparation

Sixty serum samples were collected from NMOSD-confirmed patients included in the NOMADMUS French database. Seropositive or seronegative status was established using the appropriate diagnosis criterion cell-based assay [2]. All the serum samples were collected during a first relapse of the disease in naïve patients. Sixty serum samples from naïve RRMS patients diagnosed in tertiary centers in France were obtained from the OFSEP cohort, which is a nationwide French MS registry aiming to foster research on collected standardized clinical, biological and magnetic resonance imaging data in routine. Both NMOSD and RRMS patients were treated at the acute phase of disease with corticosteroids (at least 3 g of intravenous methylprednisolone). Thirty-five samples from NEUR patients diagnosed with chronic inflammatory demyelinating polyneuropathy at the Service de Maladies inflammatoires du Système nerveux, Hôpital de Hautepierre, Strasbourg, France, were included along with 80 serum samples from HC donors purchased from the “Etablissement Français du sang” (Strasbourg, France). Demographic details of the cohort are shown in Tables S3–S6. Serum was obtained from whole blood by centrifugation at 1500× *g* for 30 min at 4 °C. The samples were flash frozen in liquid nitrogen and conserved at −80 °C until use for FTIR spectroscopy. Informed consent was obtained from all human subjects. The authors were granted approval to handle human samples by the bioethics cell of the French Ministry of Higher Education, Research and Innovation (Cellule Bioéthique-DGRI-SPFCO, Ministère de l’enseignement supérieur, de la recherche et de l’innovation, under reference: DC-2018-3209). The authors confirm that all experiments were performed in accordance with the relevant guidelines and regulations.

### 4.2. Fourier Transform Infrared Spectroscopy

The FTIR spectra of the 235 sera samples were recorded in the 4000–700 cm^−1^ spectral range using a diamond attenuated total reflection unit mounted in a Vertex 70 FTIR spectrometer (Bruker Optics, Karlsruhe, Germany). A spectral resolution of 4 cm^−1^ and a scan rate of 20 kHz were used. Each sample of 2.5 µL of serum was left to dry on the diamond surface before recording several spectra. At least 5 spectra of 64 scans each were averaged. The averaged spectra were preprocessed with OPUS 7.2 for baseline correction, normalization and generation of second derivatives. The FTIR data of HC subjects were previously published [16].

### 4.3. Statistical Analysis

In this study we built a random forest [17] classification model. It was validated using a two-fold cross validation procedure which was repeated 10 times. We used the implementation from Weka (https://www.cs.waikato.ac.nz/mL/weka/, accessed on 17 January 2022) [32], a free and open source machine learning package developed at the University of Waikato, Hamilton, New Zealand. The model consists of 100 random decision trees, all other parameters being set to default values. A random forest is a collection of random decision trees; each of the trees gives a prediction about the class of individual data, and then the trees ensemble “votes” for this classification. The forest outputs the class having the most votes among all the individual trees of the forest. During a two-fold cross-validation procedure, the dataset is split into a training set and a validation set of equal size. The training set is used to build the model that is then used to predict the samples’ class of the validation set. The procedure allows one to observe how well the model generalizes to new unseen instances. The final models are validated on a validation set that has been kept isolated from the very beginning and is used only once, to evaluate the performance of the final model.

First, a model was built to distinguish between MS, HC and NMOSD without considering the serostatus of the NMOSD patients, which means that AQP-4, MOG and DN formed a single class. To this end, the second derivatives of the 235 FTIR spectra were split into two subsets. The first subset contained 208 s derivatives (54 NMOSD, 54 RRMS, 70 HC and 30 NEUR) of the original data set. The validation set was composed of six NMOSD, six RRMS, ten HC and five NEUR.

Sensitivity, specificity, and precision were calculated according to Equations (1)–(3), respectively.

(1) $$Sensitivity\;\left(\%\right)=\frac{true\;positive\;instances}{true\;positive\;instances+false\;negative\;instances}\times 100$$

(2) $$Specificity\;\left(\%\right)=\frac{true\;negative\;instances}{true\;negative\;instances+false\;positive\;instances}\times 100$$

(3) $$Precision\;\left(\%\right)=\frac{true\;positive\;instances}{true\;positive\;instances+false\;positive\;instances}\times 100$$

## 5. Conclusions

We used a random forest classification machine learning algorithm in order to distinguish the FTIR second derivatives of serum samples from RRMS, NMOSD and NEUR patients along with serum samples from HC subjects. The NMOSD patients with three different serostatuses were also classified with the same statistical approach. First, a model was built based on a total of 202 serum samples for the discrimination between NMOSD and RRMS, HC and NEUR. The performance of the machine learning algorithm, as assessed using 10 repetitions of two-fold cross-validation, was excellent at discriminating the four groups of patients with no measurable confusion between them. Accordingly, FTIR spectroscopy coupled to a random forest classifier can offer a fast (data acquisition takes less than 30 min) and cost-effective additional tool to improve the diagnosis and differentiation of RRMS and NMOSD, regardless of serostatus. We are currently working on a Java^TM^ application automating the numerous steps (data acquisition, post-processing and machine learning classification) of the procedure across different spectrometers to take about 5 min. This Java^TM^ application will allow medical staff to perform diagnosis at point of care without the need for expertise in infrared spectroscopy or chemometrics. In addition to automating the numerous steps, we are building an algorithm that calculates a “spectrum score” and a “classification score”. The former score will guarantee the quality of the recorded spectrum and the latter score will guarantee a high degree of confidence of diagnosis for a given disease, comparatively better than current diagnostic methods.

We attempted to build a model to discriminate NMOSD patients based on their respective serostatus, but no convincing model was obtained. The small number of instances for each serostatus was insufficient to identify specific patterns in the infrared spectra of the sera, if such a signal exists at all.

## Figures and Tables

**Table 1:** Performances (confusion matrix, receiver operating characteristic curve (ROC AUC), sensitivity, specificity, and precision) of the random forest model based on second derivatives of the FTIR spectra of serum samples discriminating HC, NMOSD, RRMS and NEUR. The first row concerns two-fold cross-validation on the training set (70 HC, 54 NMOSD, 54 RRMS and 30 NEUR). The values in parentheses correspond to a ten-iteration internal validation of the model (see Materials and Methods for details). The grey rows record the performances on the test set (10 HC, 6 NMOSD, 6 RRMS and 5 NEUR). True positives are in bold and false negatives/false positives are in italics.

Pathology		Classified as				ROC AUC (%)	Sensitivity (%)	Specificity (%)	Precision (%)
		HC	NMOSD	RRMS	NEUR				
2-fold cross-validation	HC	**68**	*2*	0	0	99.8 (99.5 ± 0.5)	97.1 (99.4 ± 0.9)	98.6 (93.3 ± 5.1)	97.1 (96.7 ± 2.4)
	NMOSD	*1*	**53**	0	0	99.6 (98.7 ± 0.9)	98.1 (99.0 ± 1.3)	98.7 (78.3 ± 7.3)	96.4 (92.9 ± 2.3)
	RRMS	0	0	**54**	0	100 (100 ± 0.0)	100 (100 ± 0.0)	100 (96.9 ± 2.8)	100 (98.9 ± 0.9)
	NEUR	*1*	0	0	**29**	100 (100 ± 0.0)	100 (99.9 ± 0.3)	100 (92.7 ± 10.8)	100 (98.8 ± 1.7)
Validation set	HC	**10**	0	0	0	100	100	100	100
	NMOSD	0	**6**	0	0	100	100	100	100
	RRMS	0	0	**6**	0	100	100	100	100
	NEUR	0	0	0	**5**	100	100	100	100

**Table 2:** Performances (confusion matrix, ROC AUC, sensitivity, specificity, and precision) of the random forest model based on second derivatives of FTIR spectra of the NMOSD serum samples discriminating DN, AQP-4 and MOG. The first row concerns two-fold cross-validation on the training set (18 DN, 18 AQP-4 and 18 MOG). The values in parentheses correspond to a ten-iteration internal validation of the model (see Materials and Methods for details). The grey rows record the performances on the test set (2 DN, 2 AQP-4 and 2 MOG). True positives are in bold and false negatives/false positives are in italics.

Pathology		Classified as			ROC AUC (%)	Sensitivity (%)	Specificity (%)	Precision (%)
		DN	MOG	AQP-4				
2-fold cross-validation	DN	**10**	*4*	*4*	61.4 (69.4 ± 6.6)	55.6 (92.5 ± 8.7)	66.7 (33.3 ± 13.0)	45.5 (73.6 ± 3.6)
	MOG	*5*	**7**	*6*	58.6 (66.9 ± 7.9)	38.9 (93.1 ± 5.4)	72.2 (16.1 ± 12.2)	41.2 (69.0 ± 3.3)
	AQP-4	*7*	*6*	**5**	57.9 (61.3 ± 8.9)	27.8 (85.0 ± 9.2)	72.2 (26.1 ± 14.1)	33.3 (69.8 ± 4.3)
Validation set	DN	**1**	*1*	0	75	50	75	50
	MOG	0	**2**	0	100	100	5	66.7
	AQP-4	*1*	0	1	87.5	50	0	100

**Table 3:** Performances (confusion matrix, ROC AUC, sensitivity, specificity, and precision) of random forest model based on second derivatives of the FTIR spectra of the RRMS serum samples vs. DN NMOSD alone, vs. MOG NMOSD alone and vs. AQP-4 alone. The first row concerns two-fold cross-validation on the training set (18 DN, 18 AQP-4 and 18 MOG). The values in parentheses correspond to a ten-iteration internal validation of the model (see Materials and Methods for details). The grey rows record the performances on the test set (6 RRMS, 2 DN, 2 MOG and 2 AQP-4). True positives are in bold and false negatives/false positives are in italics.

	Pathology	Classified as		ROC AUC (%)	Sensitivity (%)	Specificity (%)	Precision (%)
		RRMS	DN				
2-fold cross-validation	RRMS	**54**	0	100 (99.9 ± 0.3)	100 (95.0 ± 6.7)	100 (98.9 ± 1.7)	100 (96.9 ± 4.8)
	DN	0	**18**	100 (99.8 ± 0.4)	100 (98.7 ± 1.8)	100 (95.0 ± 7.6)	100 (98.4 ± 2.4)
Validation set	RRMS	**6**	0	100	100	100	100
	DN	0	**2**	100	100	100	100
	**Pathology**	**Classified as**		**ROC AUC** **(%)**	**Sensitivity** **(%)**	**Specificity** **(%)**	**Precision** **(%)**
		**RRMS**	**MOG**				
2-fold cross-validation	RRMS	**54**	0	100 (99.9 ± 0.3)	100 (95.0 ± 6.7)	99.4 (98.9 ± 1.7)	98.2 (96.9 ± 4.8)
	MOG	*1*	**17**	100 (100 ± 0.0)	99.4 (95.0 ± 10.5)	100 (99.8 ± 0.8)	100 (99.5 ± 2.2)
Validation set	RRMS	**6**	0	100	100	100	100
	MOG	0	**2**	100	100	100	100
	**Pathology**	**Classified as**		**ROC AUC** **(%)**	**Sensitivity** **(%)**	**Specificity** **(%)**	**Precision** **(%)**
		**RRMS**	**DN**				
2-fold cross-validation	RRMS	**51**	*0*	99.4 (99.6 ± 0.1)	100 (93.3 ± 8.4)	83.3 (100 ± 0.0)	94.7 (100 ± 0.0)
	AQP-4	*3*	**15**	99.4 (99.9 ± 0.1)	83.3 (100 ± 0.0)	100 (91.9 ± 9.9)	100 (97.2 ± 3.0)
Validation set	RRMS	**6**	0	100	100	100	100
	AQP-4	0	**2**	100	100	100	100

## Supplementary Materials

The following supporting information can be downloaded at: https://www.mdpi.com/article/10.3390/ijms23052791/s1. Refs. [18,20,21,34] are cited in the Supplementary Materials.
