# Supplementary material for: Rapid Discrimination of Neuromyelitis Optica Spectrum Disorder and Multiple Sclerosis Using Machine Learning on Infrared Spectra of Sera
Source: Int J Mol Sci. 2022 Mar 3;23(5):2791. doi: 10.3390/ijms23052791 (PMC8911153; doi:10.3390/ijms23052791)
Supplement: Supplementary file 1 [file ijms-23-02791-s001.zip › ijms-1586266-supplementary.pdf]

SUPPORTING INFORMATION

Rapid discrimination of neuromyelitis optica spectrum disorder and multiple sclerosis using machine learning on infrared spectra of sera

Youssef El Khoury[1,*], Marie Gebelin[1], Jérôme de Sèze[,3], Christine Patte-Mensah[2], Gilles Marcou[4], Alexandre Varnek[4], Ayikoé-Guy Mensah-Nyagan[2,$], Petra Hellwig[1,$], and Nicolas Collongues[2,3,5 *]

[1] Laboratory of Bioelectrochemistry and Spectroscopy, UMR 7140 University of Strasbourg, CNRS,
4 Rue Blaise Pascal, 67000 Strasbourg, France; marie.gebelin@etu.unistra.fr (M.G.); hellwig@unistra.fr (P.H.)

[2] Biopathology of Myelin, Neuroprotection and Therapeutic Strategies, INSERM U1119, Federation of
Translational Medicine of Strasbourg, Université of Strasbourg. 1, Rue Eugène Boeckel, 67000 Strasbourg, France; Jerome.deseze@chru-strasbourg.fr (J.d.S.); cmensah@unistra.fr (C.P.-M.);
gmensah@unistra.fr (A.-G.M.-N.)

[3] Department of Neurology, University Hospital of Strasbourg, 1 Avenue Molière, 67200 Strasbourg, France

[4] Laboratory of Chemoinformatics, UMR 7140 University of Strasbourg/CNRS, 4 Rue Blaise Pascal,
67000 Strasbourg, France; g.marcou@unistra.fr (G.M.); varnek@unistra.fr (A.V.)

[5] University Department of Pharmacology, Addictology, Toxicology and Therapeutic, University of Strasbourg, 67000 Strasbourg, France

[$] Co-seniority for A.-G.M.-N. (Head of INSERM U1119) and P.H. (Head of UMR 7140)

[*] Correspondence: elkhoury@unistra.fr (Y.E.K.); nicolas.collongues@chru-strasbourg.fr (N.C.)

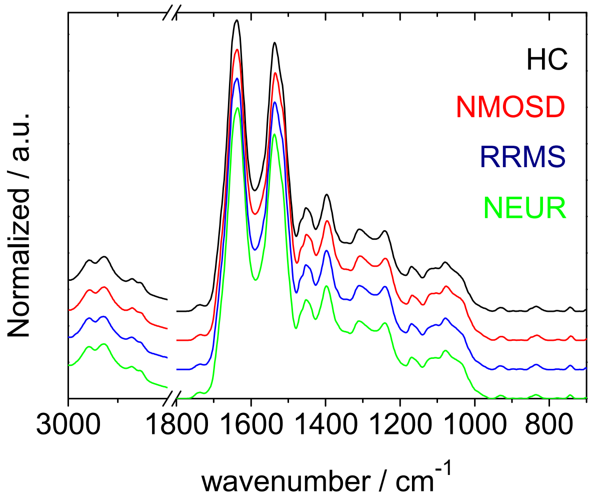

Figure S1. Normalized average spectra of the HC samples (black), NMOSD (red), RRMS (blue) and NEUR (green) in the 3000-2800, 1800-700 cm^{-1} spectral range.

Table S1. Assignments[1-4] of the FTIR signals of the averaged spectra shown in **Fig. S1**. The position of the maxima in cm^{-1} is given for the HC averaged spectrum. The maximal deviation of the peaks in the average spectra of NMOSD, RRMS and NEUR relative to the peaks' maxima found in the HC spectrum is indicated in parentheses.

Position / cm^{-1}	Biomolecule
2958 (±-1)	Lipids and proteins
2929 (±-1)	Lipids and proteins
2871 (±-1)	Lipids and proteins
2852 (±-1)	Lipids and proteins
1738 (±-2)	Lipids (Ester C=O)
1640 (±-4)	Proteins
1537 (±-2)	Proteins
1453 (±-1)	Lipids and proteins
1397 (±-1)	Lipids and proteins
1307 (±-1)	Proteins
1240 (±-2)	DNA
1169 (±-2)	Glycoproteins and carbohydrates
1117 (±-3)	RNA
1104 (±-1)	Carbohydrates
1080 (±-2)	Carbohydrates, DNA, RNA
1031 (±-1)	Carbohydrates and DNA
929 (±-3)	Left-handed DNA (Z-form)
834 (±-2)	DNA (B and Z forms)
746 (±-2)	DNA

Table S2. Performances (confusion matrix, ROC AUC, sensitivity, specificity, and precision) of a randomly resampled dataset (with equal class sizes) from the original data set used to produce Table 1 of the main text. The first row concerns two-fold cross-validation on the training set (30 HC, 30 NMOSD, 30 RRMS and 30 NEUR). The values in parentheses correspond to a ten-iteration internal validation of the model. The dark gray rows record the performances on the original validation set (10 HC, 6 NMOSD, 6 RRMS and 5 NEUR) and the light gray rows record the performances on a randomly resampled validation set with equal class sizes (5 HC, 5 NMOSD, 5 RRMS and 5 NEUR). True positives are in bold and false negatives/false positives are in italics.

**Pathology**		**Classified as**				ROC AUC (%)	Sensitivity (%)	Specificity (%)	Precision (%)
		HC	NMOSD	RRMS	NEUR				
**2-fold cross-validation**	HC	**27**	*3*	0	0	98.9 (99.7±0.3)	90.0 (94.0±6.8)	97.8 (99.1±1.8)	93.1 (97.6±4.8)
	NMOSD	*2*	**28**	0	0	99.1 (96.6±0.4)	93.3 (96.5±5.5)	96.7 (89.4±15.2)	90.3 (95.7±5.0)
	RRMS	0	0	**30**	0	100 (100±0.0)	100 (100±0.0)	100 (100±0.0)	100 (100±0.0)
	NEUR	0	0	0	**30**	100 (100±0.0)	100 (98.8±1.0)	100 (97.7±3.9)	100 (99.2±1.3)
**Validation set**	HC	**10**	0	0	0	100	100	100	100
	NMOSD	0	**6**	0	0	100	100	100	100
	RRMS	0	0	**6**	0	100	100	100	100
	NEUR	0	0	0	**5**	100	100	100	100
**Validation set**	HC	**5**	0	0	0	100	100	100	100
	NMOSD	0	**5**	0	0	100	100	100	100
	RRMS	0	0	**5**	0	100	100	100	100
	NEUR	0	0	0	**5**	100	100	100	100

Table S3. Demographic details of the NMOSD patients participating in the study, including the confirmed serostatus as well as the age at which the serum sample was taken.

ID: patient ID. Gender: male (M) or female (F). 26 M/34 F

Age: age at sample collection. Median age ± standard deviation=32.0 ± 18.4 years.

Delay: Delay in days between episode and sample collection. The median ± standard deviation delay between the relapse and the sample collection for NMOSD patients was 20±19 days for anti-AQP-4-positive patients, 25±32 days for anti-MOG-positive patients and 176±1287 days for DN patients. Median delay ± standard deviation=27 ± 863 days.

ID	Gender	Age	serostatus	Delay	ID	Gender	Age	serostatus	Delay
**1**	F	25	AQP-4	13	**31**	F	24	MOG	25
**2**	F	52	AQP-4	22	**32**	F	21	MOG	25
**3**	F	56	AQP-4	17	**33**	F	13	MOG	75
**4**	M	80	AQP-4	8	**34**	M	43	MOG	43
**5**	F	55	AQP-4	13	**35**	F	14	MOG	NA
**6**	F	67	AQP-4	7	**36**	F	6	MOG	11
**7**	F	25	AQP-4	24	**37**	M	16	MOG	9
**8**	F	62	AQP-4	2	**38**	M	6	MOG	38
**9**	F	29	AQP-4	19	**39**	M	13	MOG	6
**10**	F	55	AQP-4	21	**40**	M	7	MOG	123
**11**	M	40	AQP-4	21	**41**	F	26	DN	129
**12**	F	17	AQP-4	41	**42**	F	49	DN	47
**13**	F	32	AQP-4	15	**43**	M	33	DN	21
**14**	F	37	AQP-4	27	**44**	F	52	DN	63
**15**	M	24	AQP-4	47	**45**	F	47	DN	6
**16**	F	63	AQP-4	48	**46**	M	62	DN	5
**17**	F	44	AQP-4	67	**47**	M	18	DN	3613
**18**	F	65	AQP-4	16	**48**	M	61	DN	2267
**19**	F	31	AQP-4	64	**49**	M	58	DN	3563
**20**	F	53	AQP-4	8	**50**	F	22	DN	46
**21**	F	53	MOG	72	**51**	M	69	DN	764
**22**	M	32	MOG	6	**52**	F	29	DN	58
**23**	M	38	MOG	4	**53**	M	18	DN	10
**24**	M	26	MOG	17	**54**	F	35	DN	1168
**25**	M	53	MOG	6	**55**	F	22	DN	2766

**26**	M	36	MOG	49	**56**	M	25	DN	2088
**27**	M	26	MOG	15	**57**	F	30	DN	52
**28**	M	29	MOG	52	**58**	F	29	DN	238
**29**	M	30	MOG	63	**59**	M	45	DN	2316
**30**	M	73	MOG	11	**60**	F	25	DN	222

Table S4. Demographic details of the RRMS patients participating in the study, including the age at which the serum sample was taken.

ID: patient ID; Gender: male (M) or female (F). 19 M/41 F; Age: age at sample collection. Mean age 31.5±9.4 years; EDSS: Patient's Expanded Disability Status Scale score; Delay 1: Delay in days between episode and EDSS; Delay 2: Delay in days between episode and sample collection. Median delay 2 ± standard deviation=21± 97 days.

ID	Gender	age	EDSS	Delay 1	Delay 2	ID	Gender	age	EDSS	Delay 1	Delay 2
**1**	M	31	0.0	49	37	**31**	F	22	0.0	111	112
**2**	M	23	3.0	16	19	**32**	F	28	3.0	6	11
**3**	F	36	2.0	6	10	**33**	F	22	1.0	13	14
**4**	F	22	2.0	32	32	**34**	F	48	1.5	36	33
**5**	M	24	3.0	3	3	**35**	M	35	0.0	3	7
**6**	F	47	1.0	74	74	**36**	F	22	0.0	153	154
**7**	M	21	1.0	10	13	**37**	F	40	2.5	249	245
**8**	F	19	1.5	111	93	**38**	F	33	2.0	11	11
**9**	M	33	1.0	16	19	**39**	M	36	0.0	33	33
**10**	F	18	2.0	12	15	**40**	M	29	2.0	107	2
**11**	M	22	2.0	15	16	**41**	M	33	3.0	10	10
**12**	F	24	2.0	13	18	**42**	M	39	2.0	69	21
**13**	F	39	2.0	11	8	**43**	F	22	2.0	35	7
**14**	F	42	1.0	86	86	**44**	F	36	2.0	27	27
**15**	M	28	1.5	824	593	**45**	F	37	1.5	76	57
**16**	F	19	3.0	2	4	**46**	M	40	2.0	76	25
**17**	F	42	1.0	5	5	**47**	F	37	1.5	109	21
**18**	F	37	3.0	4	11	**48**	F	38	0.0	12	9
**19**	F	34	1.0	0	22	**49**	F	25	3.0	34	29
**20**	F	50	2.0	58	58	**50**	F	19	4.0	14	12
**21**	F	44	1.0	17	17	**51**	F	24	4.0	35	32
**22**	M	37	0.0	36	42	**52**	M	33	3.0	62	59

**23**	F	31	1.5	116	119	**53**	M	18	4.0	401	394
**24**	F	22	1.0	20	22	**54**	F	24	2.0	7	5
**25**	M	29	1.5	27	27	**55**	M	48	2.0	43	41
**26**	F	24	1.0	0	2	**56**	F	37	2.0	157	155
**27**	F	22	3.0	5	9	**57**	F	29	5.0	0	1
**28**	F	31	2.0	160	159	**58**	F	23	2.5	42	43
**29**	F	47	1.0	17	17	**59**	F	32	2.0	14	18
**30**	M	59	1.5	150	150	**60**	F	33	2.0	19	16

Table S5. Demographic details of the NEUR patients participating in the study. All patients suffered from chronic inflammatory demyelinating polyneuropathy. Serum samples were collected at least one month after the patient stabilized without immune globulin intravenous (IgIv) injection.

ID: patient ID; Gender: male (M) or female (F). 25 M/10 F; Age at sample collection; Median age ±standard deviation=59.0 ± 12.3 years.

ID	Gender	Age
**1**	M	64
**2**	M	51
**3**	F	70
**4**	M	75
**5**	M	66
**6**	F	54
**7**	M	57
**8**	M	44
**9**	F	66
**10**	M	80
**11**	M	55
**12**	M	50
**13**	M	72
**14**	F	69
**15**	M	61
**16**	M	59
**17**	F	51
**18**	M	59
**19**	M	59

**20**	M	53
**21**	M	83
**22**	F	66
**23**	F	82
**24**	M	56
**25**	M	43
**26**	F	58
**27**	F	64
**28**	M	78
**29**	F	48
**30**	M	67
**31**	M	24
**32**	M	68
**33**	M	57
**34**	M	54
**35**	M	51

Table S6. Demographic details of the HC subjects participating in the study, including the age at which the serum sample was taken; 80 subjects with 1:1 ratio M/F and 1:1 ratio of subjects younger/older than 60 years.

Gender	Number of subjects	Younger than 60 years	Older than 60 years
M	40	20	20
F	40	20	20

Figure S2. ROC curves of the four patient groups used for the single-iteration of the random forest model presented in Table 1 of the main text. HC (black), NMOSD (red), RRMS (blue) and NEUR (green).

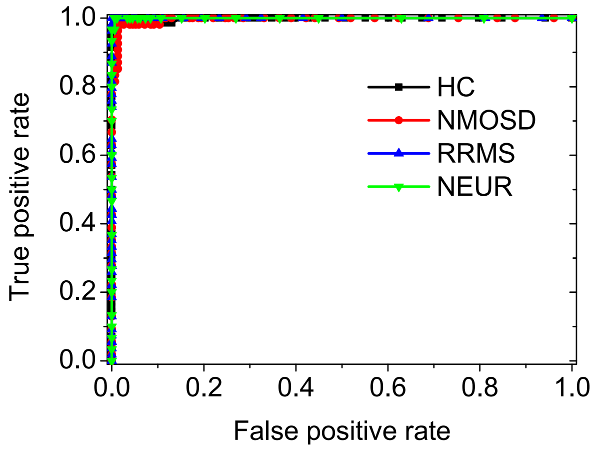

Figure S3. ROC curves of NMOSD patients according to their confirmed serostatus. DN (dark red), AQP-4 (violet), and MOG (light red).

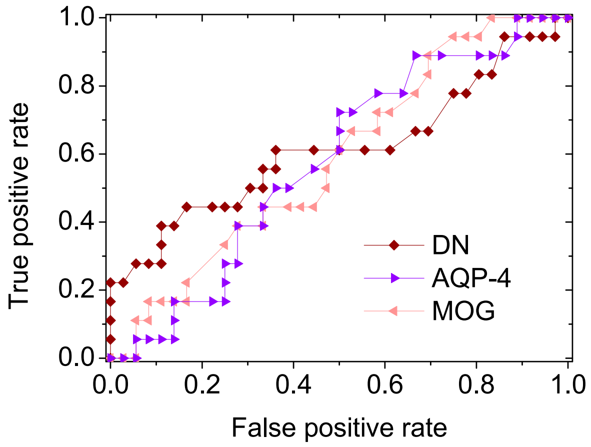

Figure S4. ROC curves of RRMS and NMOSD patients according to their confirmed serostatus. A) RRMS and DN; B) RRMS and AQP-4; C) RRMS and MOG. RRMS (blue squares), DN (dark red circles), AQP-4 (violet circles) and MOG (light red circles).

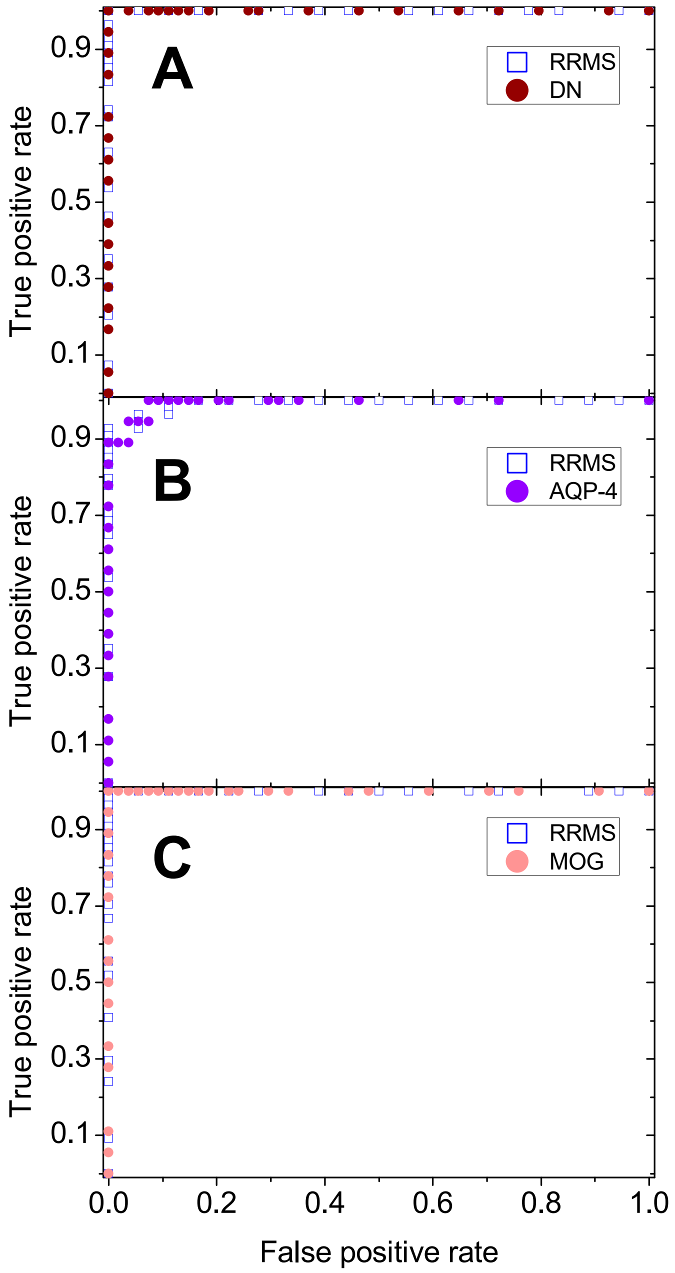

References

1. Rehman, I.U.; Movasaghi, Z.; Rehman, S. *Vibrational spectroscopy for tissue analysis*, 1st Edition ed.; CRC Press: Boca Raton, **2012**.
2. Ghomi, M.; Letellier, R.; Liquier, J.; Taillandier, E. Interpretation of DNA vibrational spectra by normal coordinate analysis. *Int. J. Biochem.* **1990**, *22*, 691-699.
3. Baker, M.J.; Trevisan, J.; Bassan, P.; Bhargava, R.; Butler, H.J.; Dorling, K.M.; Fielden, P.R.; Fogarty, S.W.; Fullwood, N.J.; Heys, K.A.; *et al.* Using Fourier transform IR spectroscopy to analyze biological materials. *Nat. Protoc.* **2014**, *9*, 1771-1791, doi:10.1038/nprot.2014.110.
4. Barth, A. Infrared spectroscopy of proteins. *Biochim. Biophy. Acta* **2007**, *1767*, 1073-1101, doi:10.1016/j.bbabio.2007.06.004.
